# The depleted mineralization of the fungicide chlorothalonil derived from loss in soil microbial diversity

**DOI:** 10.1038/s41598-017-14803-0

**Published:** 2017-11-07

**Authors:** Adijailton Jose de Souza, Pedro Avelino Maia de Andrade, Arthur Prudêncio de Araújo Pereira, Fernando Dini Andreote, Valdemar Luiz Tornisielo, Jussara Borges Regitano

**Affiliations:** 1 Soil Microbiology Laboratory, Soil Science Department, Luiz de Queiroz College of Agriculture, University of São Paulo, Piracicaba, Brazil; 2 Ecotoxicology Laboratory, Center for Nuclear Energy in Agriculture, University of São Paulo, Piracicaba, Brazil

## Abstract

There are lack of studies regarding the effects of microbial diversity on specific soil functions, such as pesticides degradation. This study evaluated the role of bacterial community diversity and biochar on chlorothalonil (CTN) degradation, using ‘dilution to extinction’ approach, PCR-DGGE/16S rRNA gene technique, and radiorespirometry (^14^C-CTN). Biochar and microbial community dilution affected structure of the microbial community. In spite of that, CTN mineralization was slow, but dissipation was very fast (D_50_ < 1.0 d) due to immediate chemical degradation and formation of non-extractable (bound) residues. However, any depletion on soil microbial diversity strongly affected CTN mineralization, suggesting that this function is related to less abundant but specific microbial groups (CTN degraders) or to soil microbial diversity. The extent of these effects will strongly depend on the compound nature (recalcitrance) and soil matrix/substrate (bioavailability). It can be corroborated by the fact that biochar affected CTN sorption, its bioavailability, and subsequently its mineralization rate in the NS. These data indicate a strong relationship between soil microbial diversity and pesticide degradation, which is an acting form to mitigate xenobiotics accumulation in the environment.

## Introduction

Despite having a central role in the Earth’s biogeochemical cycles, microbial diversity studies have been neglected until the last decade^1,2^. The central issue regarding biodiversity and ecosystem functioning (BEF, abbreviation) involves how depletion and/or decline in species diversity affect essential ecosystem functioning processes^3–6^. It is well known that greater species diversity is needed to ensure better ecosystem performance^7,8^. In this way, several works has shown the existence of a positive correlation between species richness and ecosystem functioning^7,9,10^. For more specific functions, the ecosystem response may not depend on microbial community diversity^1,11^. As a matter of fact, functional redundancy should be greater for processes that are continuous and intense in the ecosystem, which should be carried out by distinct microbes under different environmental conditions. Examples are carbon mineralization, biomass accumulation, and resource efficiency, which tend to be less affected by loss of microbial diversity^10,12^. However, specific functions can be achieved by particular microbes under specific environmental conditions^13,14^, such as nitrification and methane oxidation, which tend to be more affected by diversity loss^3,15^. Therefore, it is important to determine whether changes in microbial diversity can impact essential ecosystem services^16–18^, especially with regard to their more specific functions, such as pesticides degradation.

Pesticides degradation are fundamental in mitigating their deleterious effects on the environment. The vast majority of these compounds comprehends organic molecules, which can be degraded microbiologically, usually reducing their toxicity^19^. Therefore, soil biodegradation of pesticides will depend either on the microbial diversity or on the presence of specific groups capable of degrading the compound, which, in turn, will depend on pesticide bioavailability and on environmental conditions. In general, the higher the pesticide sorption to soil particles, the lower its bioavailability and, consequently, the lower its biodegradation rate^20^.

Bioavailability is a crucial factor dictating environmental fate of pesticides, as it directly interferes with their persistence and degradation rate^21,22^. Several soil conditioners may influence xenobiotics bioavailability in the environment. Currently, biochar, a stable carbon rich material obtained from biomass pyrolysis, has raised great interest as a soil conditioner due to enhancing carbon sequestration and pollutants sorption (retention); as well as improving physical, chemical, and biological soil properties^23,24^. Carbonaceous compounds, such as biochar, act as efficient adsorbent agents for several contaminants due to their high surface area (SSA) and high aromaticity (i.e. high amounts of hydrophobic sites)^22,25^.

The main objective of this study was to evaluate the effect of soil biodiversity on the degradation of pesticides, using the CTN fungicide and the dilution to extinction technique as models. In parallel, biochar was added to the system in order to evaluate its impact on microbial diversity, on pesticide bioavailability, and subsequently on degradation rate of CTN. CTN (a broad-spectrum, non-systemic organochloride fungicide) was selected due to its extensive use in several crops in Brazil^26,27^. It has low solubility (S_w_ = 0.85 mg L^−1^ at 25 °C), low to moderate persistence (DT_50_ = 5 to 90 d), high sorption (K_oc_ = 5000 L Kg^−1^), low mobility^27,28^, and microbial degradation as its main dissipation route in soils. In addition, CTN degradation involves either displacement of a chlorine by a hydroxyl group forming 4-hydroxy-2,5,6-trichloroisophthalonitrile or oxidation/hydration of a cyano to a corresponding amide and an organic acid group forming 3-cyano-2,4,5,6-tetrachlorobenzamide and 3-carbamyl-2,4,5-trichlorobenzoic acid^29–31^. This last metabolite was the most abundant in Brazilian soils, corresponding to 18–25% of the applied amount, whereas the first one was most abundant in other scenarios (mostly at temperate conditions)^32^, which causes environmental concerns since it is more acutely toxic (30 times), persistent, and mobile than CTN itself ^27,29^.

## Results

### Dilution and biochar effects on soil bacterial community

The soil community dilution promoted artificial modification in the microbial diversity of the natural soil (NS) (Supplementary Fig. S1). Diluted soils incubation for 15 d was enough to allow differentiation among communities’ structures by the adopted technique (PCR-DGGE) (Supplementary Figs S1 and S2). This diversity gradient allows to properly assessing interactions between community structures, biochar role, and pesticides biodegradation.

**Figure 1:**
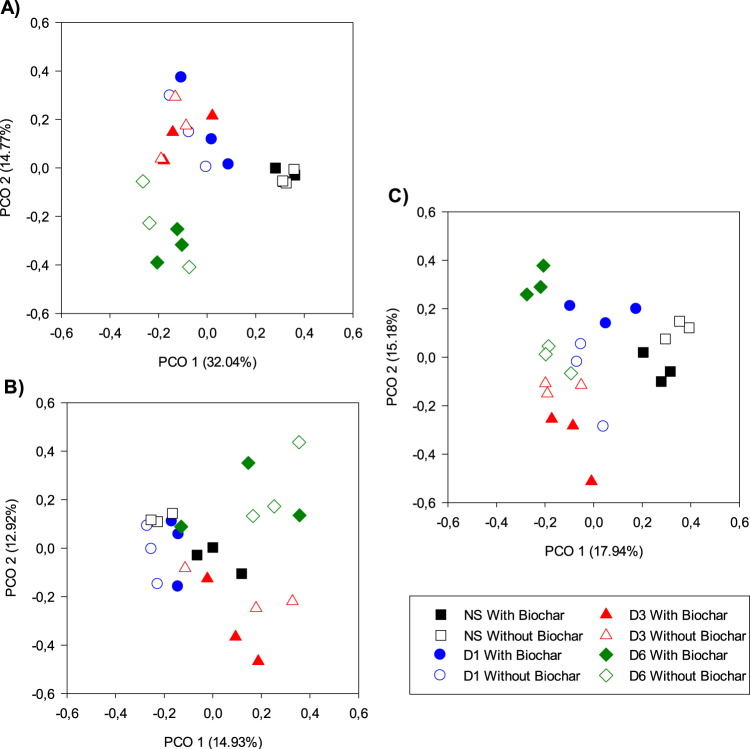
Principal coordinate analysis (PCO) of the bacterial community profile (DNA bands) after microbial dilution, chlorothalonil and biochar application in the soil. Legends: A = 1 day after application; B = 21 days after application; C = 42 days after application; NS = natural soil (control); D1 = dilution 10^−1^; D3 = dilution 10^−3^ and D6 = dilution 10^−6^.

**Figure 2:**
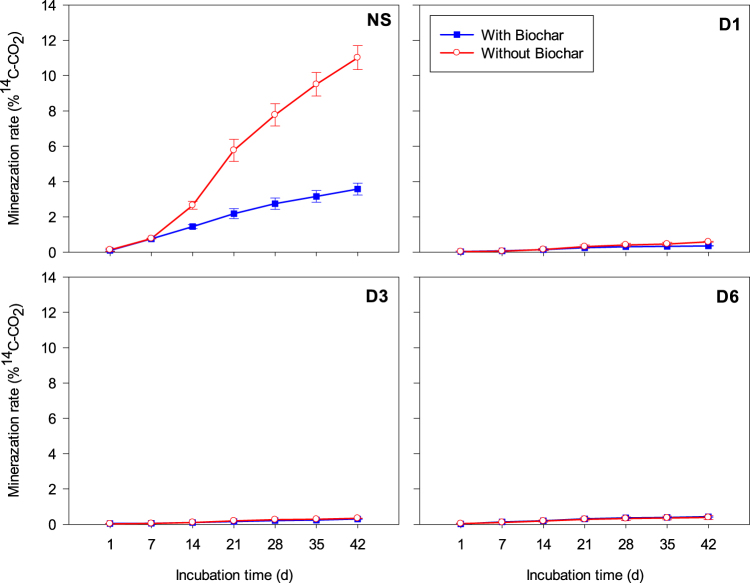
Mineralization rate (^14^CO_2_ evolved) of chlorothalonil in soil after microbial dilution and biochar application during 42 d. Legends: NS = natural soil (control); D1 = dilution 10^−1^; D3 = dilution 10^−3^ and D6 = dilution 10^−6^. Standard error bars (n = 2).

After biochar and CTN application, the bacterial community was structured in distinct clusters throughout the different sampling periods (1, 21, and 42 d), validated by the R values (R = 0.83 to 0.87 and p < 0.001) that were calculated by the logarithm of Bray Curtis (Supplementary Table S1). Initially (at 1 d), diluted bacterial communities were maintained in distinct clusters, but no differentiation was observed between the treatments with and without biochar (Figs 1A and 2A). Subsequently, at 21 d, there was greater dispersion of bacterial communities, and biochar addition started to affect their structuring process (Fig. 1B). After 42 d, it is evident that biochar changed the structure of the bacterial communities in the different dilutions (Fig. 1C), which were also distinct among themselves. Apart of biochar addition, the initial profile and number of DNA bands (16S rRNA gene) in the diluted (10^−1^, 10^−3^, and 10^−6^) samples were lower than in the NS (Supplementary Figs 3A and 4A), but it recovered later, *i.e*. at 21 and 42 d (Supplementary Fig. 3B,C and 4B,C).

### Biochar effect on CTN sorption

Soil sorption of CTN was high (>5 L kg^−1^) and enhanced in the presence of biochar, independently of its sizing (Table 1). For example, the sorption coefficient (K_d_^ap^) increased from 17.3 to 34.5 L kg^−1^ after fine (triturated) biochar application. These values corresponded to a sorption of 76 and 87% of the CTN applied amounts, respectively.

**Table 1:** Percentages sorbed (S) and apparent sorption coefficients (K_d_^ap^) of chlorothalonil in the natural soil and after differently textured biochar applications.

Treatment	S (%)	K_d_^ap^ (L kg^−1^)
Control (without biochar)	76.4	17.3
Fine biochar (<2.0 mm)	86.6	34.5
Medium biochar (2.0 mm)	82.3	24.8
Unsieved biochar	84.6	29.3

### Dilution and biochar effects on CTN degradation

CTN mineralization rates, as determined by^14^CO_2_ release, were lower in the diluted treatments (D_1_, D_2_, and D_3_ < 0.6% of the applied amount) than in the NS (= 3.6 and 11.0% in the soil with and without biochar, respectively) (Fig. 2), showing that biochar also inhibited CTN mineralization (Fig. 2).

**Figure 3:**
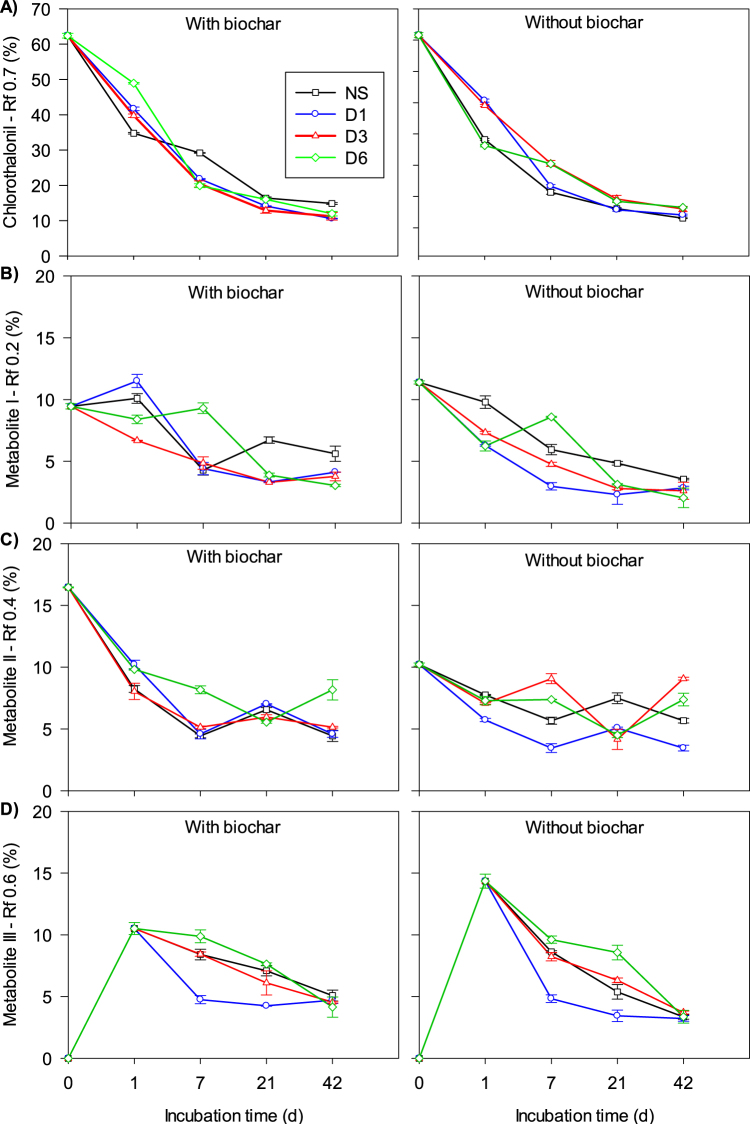
Chlorothalonil dissipation and metabolites formation after microbial dilution and biochar application up to 42 d. Legends: A = Chlorothalonil; B = Metabolite I; C = Metabolite II; D = Metabolite III; NS = Natural Soil (Control); D1 = Dilution 10^−1^; D3 = Dilution 10^−3^; D6 = Dilution 10^−6^. Standard error bars (n = 2).

**Figure 4:**
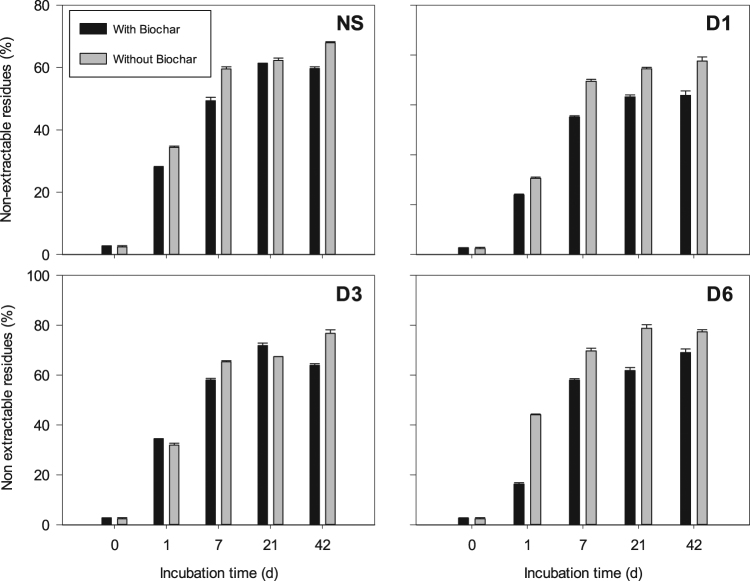
Non-extractable residues (bound residue) of chlorothalonil after different microbial dilution and biochar application during 42 d. Legends: NS = Natural Soil (Control); D1 = Dilution 10^−1^; D3 = Dilution 10^−3^; D6 = Dilution 10^−6^. Standard error bars (n = 2).

Despite the slow mineralization, CTN exhibited very fast dissipation rate (Fig. 3A). Its half-life values (DT_50_) were less than 1 d for all treatments. For the NS without biochar, the DT_90_ values were equal to 10, 13, 19, and 20 d in the control and 10^−1^, 10^−3^, and 10^−6^ dilutions, respectively (Fig. 3A). Immediately after application (t = 0 d), only ~62% of the applied CTN was recovered (Fig. 3A). It suggests that the molecule was quickly and chemically degraded to two unidentified metabolites (M-I and M-II), in percentages ranging from 9.5 to 16.5% (Fig. 3B,C). A third metabolite (M-III) was detected 1 d after application (Fig. 3D). After 42 d, only 3–15% of the applied CTN was recovered (Fig. 3A). Furthermore, the formation of non-extractable (“soil bound”) residues was fast and in high amounts (Fig. 4), contributing significantly to the fast dissipation of CTN. In all treatments, this fraction corresponded to more than 50% of the CTN-applied amount at 7 d and was considerably higher for the diluted treatments (Fig. 4).

## Discussion

Microbial diversity depletion can be reached by different approaches, such as defined mixture of microbe species, fumigation, and dilution to extinction^16,33^. The first one considers only the cultivable fraction of the microbial community, representing a little more than 1% of the total community and, therefore, being little representative of natural ecosystems. The second one (fumigation) showed inconsistent relationships between diversity and ecosystem functioning^34,35^. Moreover, this methodology does not allow constructing a diversity gradient since it promotes a non-selective effect on microorganisms^16^. This work used the validated ‘dilution to extinction’ method in order to evaluate the effect of diversity depletion on pesticide degradation.

Other studies, based on the same approach, suggested that a time period is needed in order that the modified soil microbial community could reestablish in terms of cell density or microbial biomass^3,35–38^. In our case, this period took 15 d and it was set as the starting point for experimental trials (Fig. S2). The use of this method mimics soil biodiversity degradation due to environmental pollution, or non-conservative agricultural practices, providing indications about the loss of environmental services prompted by changes in the microbial diversity of these modulated scenarios.

Our results showed that both microbial community dilution and biochar application changed microbial community structure. Possibly, these drivers act in combination since dilution gradient results in loss of soil microbial diversity and biochar provides new niches/sites for microbial colonization^39,40^ and pesticides sorption^41,42^. Both processes for biochar (protection and sorption) should decrease molecules availability to soil microbes, affecting their structure and pesticide biodegradation. Biochar application usually enhances physical, chemical, and biological properties of the soil as well as its capacity to sorb different pollutants^24,41,42^. Furthermore, biochar can provide surface and internal niches for microbial colonization due to the presence of volatile compounds that can be used as a source of C and provide protection against desiccation and predation^39^. Biochar may also reduce bioavailability of organic and inorganic contaminants, which may affect soil microbial community^43,44^. For last, the presence of CTN seemed have not affected the structure of soil bacterial community. Similar results were attained CTN and other fungicides, such as azoxistrobin and tebuconazole^43,45^.

Soil microbial community plays important roles in crucial processes of the soil, such as maintenance of biogeochemical cycles, decomposition of organic matter, biological nitrogen fixation, nutrient solubilization, and removal of pollutants. Hence, changes in microbial diversity induced here will impact essential ecosystem functions^16–18^, but should also affect its more restrict functions, such as pesticide degradation. Actually, in spite of the overall low mineralization rate of CTN, it was greatly affected by the dilution of soil microbial community due to either the extinction of active degrader groups or the decrease in soil biodiversity that would lead to a lower microbial activity, which may be triggered by the diminished functional redundancy in the soil. In other words, any small change in the dilution factor in relation to the original community (NS) strongly reflected in reduction of CTN mineralization rate. Other studies, using the same approach (dilution to extinction), showed that the evaluated ecological functions were partially or totally lost only at higher dilution factors (>10^−6^)^16,36^. Hernandez-Raquet *et al*.^36^ observed that microbial community from activated sewage sludge partially lost the ability to mineralize phenanthrene at dilution factors above 10^−3^, but its mineralization completely ceased at much higher dilution factors (10^−5^ and 10^−8^). Our results suggest that any minor change either on the structure of microbial community or on the diversity of microbial population strongly affected CTN mineralization rate, showing that this process is very restrict to specific degraders, whereas it does not seem to be the case for phenanthrene. In summary, soil microbial diversity will affect more restrict functions, such as xenobiotic degradation, but the extent of these effects will depend on pesticide type and on substrate nature^11,19^. In general, more recalcitrant molecules and soils with higher sorption potential (i.e. soils having lower amounts of available pesticides) should be less affected by soil microbial diversity^36,46^.

The presence of biochar decreased mineralization rate of CTN only in the NS, but it had no effect in the diluted treatments since microbial population showed restricted mineralization capacity. The lower mineralization rate in the NS with biochar is likely due to the higher sorption potential and, consequently, the lower bioavailability of CTN since biochar had little influence on soil microbial community structure initially. Different authors have highlighted the sorbing properties of biochar for different classes of pesticides in soils^42,47–49^.

CTN exhibited high dissipation rate. Only ~62% of the applied CTN was recovered just after application. This immediate dissipation corroborates to the fact that the initial degradation of the CTN is primarily chemical, although microbial degradation also plays its role afterwards. The fact that DT_90_ values were lower for the treatment with the highest diversity (NS, 10 d), but higher for the treatment with the smallest diversity (D6, 20 d) reinforces the role of microbial population on CTN degradation. In different conditions, other authors have already reported fast dissipation rates for this fungicide^32,50^. CHAVES *et al*.^26^ pointed out that 44% of the applied CTN was dissipated in the first hours after its application in a soil cultivated with banana. It is convenient to point out that one of the CTN metabolites, the 4-hydroxy-2,5,6-trichloroisophthalonitrile, commonly found under temperate conditions, may be more toxic (lower LD_50_) and susceptible to leaching than the parent molecule, which may exacerbate environmental problems^29,51^. Although the presence of these byproducts may pose a threat to the environment and non-target organisms, their concentrations at the end of the experiment were low (<10%) and should not cause major problems. The formation of non-extractable residues constituted an important route of dissipation, reinforcing results from previous studies^32,52,53^. The authors observed strong interactions between CTN and the organic fraction of the soil, reflecting its high sorption potential and, therefore, its low mobility in soils^27^.

## Conclusion

CTN has fast dissipation rate due primarily to its immediate chemical degradation and formation of non-extractable (bound) residues. Removal of microbial community diversity, by dilution, drastically affects CTN mineralization rate, thus compromising this microbial function of the ecosystem. The use of biochar affects CTN bioavailability, diminishing its mineralization rate. However, it does not affect population density, even in the natural soil. The extension of the microbial diversity effects on pesticides degradation will strongly depend on the compound nature and soil matrix (substrate). Further studies, with the same approach, but considering different classes of compounds would be essential to better clarify those effects.

## Material and Methods

A NS sample of a Ferralsol Haplic was collected from 0 to 10 cm layer of a native area without pesticide application history, which was sieved (2 mm) and stored at 4 °C for 3 d (detailed methodology, item 1).

The experiments were performed under controlled conditions, developed in microcosms (Bartha’s flasks, 250 ml). Each experimental unit consisted of 25 g of air-dry soil samples, which were autoclaved (1 atm, 120 °C, 3 cycles of 1 h) and re-inoculated with microbial dilutions of 10^−1^, 10^−3^ e 10^−6^, adopting the ‘dilution to extinction’ methodology and the NS as a control (detailed methodology, item 2). After restructuring the microbial community, biochar (10.0 t ha^−1^ or 1.0% w/w) and CTN (1.8 kg ha^−1^ or 1.385 μg g^−1^) were applied to the different treatments.

Soil microbial restructurings were evaluated during 15 d whereas changes on microbial communities were evaluated at 1, 21, and 42 d after biochar and CTN application. Therefore, ~2.0 g of soil subsamples of each treatment were collected in order to extract total DNA and verify its quality by agarose gel electrophoresis. The DNA was submitted to polymerase chain reactions (PCR) for amplification of the 16 S rRNA gene. The DNA fragments amplified in the first reaction were then submitted to the PCR-DGGE reaction and the band-profiles counted on gel electrophoresis (detailed methodology, item 3).

In parallel, and simultaneously, the same experiments were conducted using radiolabeled molecule (^14^C-CTN) (detailed methodology, item 2) in order to evaluate its mineralization, dissipation, and metabolism rates, as well as formation of non-extractable (bound) residues (detailed methodology, items 4, 5, 6). These parameters were evaluated 0, 1, 7, 21, and 42 d after biochar and pesticide application.

^14^C-CTN sorption to the NS after sized-biochar application was also evaluated (detailed methodology, items 7).

### Data Availability Statement

The authors of the manuscript “The depleted mineralization of the fungicide chlorothalonil derived from soil microbial diversity” state that all data generated or analyzed during this study are included in this published article (and its Supplementary Information files).

## Electronic supplementary material

Supplementary Information
