# Supplementary material for: The depleted mineralization of the fungicide chlorothalonil derived from loss in soil microbial diversity
Source: Sci Rep. 2017 Nov 7;7:14646. doi: 10.1038/s41598-017-14803-0 (PMC5676787; doi:10.1038/s41598-017-14803-0)
Supplement: Supplementary file 1 — Supplementary Information [file 41598_2017_14803_MOESM1_ESM.doc]

Supplementary information

**The depleted mineralization of the fungicide chlorothalonil derived from loss in soil microbial diversity**

Adijailton Jose de Souza^1^; Pedro Avelino Maia de Andrade^1^; Arthur Prudêncio de Araújo Pereira^1^; Fernando Dini Andreote^1^; Valdemar Luiz Tornisielo^2^; Jussara Borges Regitano^1*^

Author Affiliations:

^1^ Soil Microbiology Laboratory, Department of Soil Science, Luiz de Queiroz College of Agriculture, University of São Paulo, Piracicaba, Brazil

^2^ Ecotoxicology Laboratory, Center for Nuclear Energy in Agriculture, University of São Paulo, Piracicaba, Brazil

*Correspondence author: regitano@usp.br

**This file includes:**

Supplementary figures S1, S2, S3, and S4

Supplementary table 1

**Supplementary Figure S1. Principal coordinate analysis (PCO) of the bacterial community profile (DNA bands) during initial structuring (15 d).** Legends: A = immediately after dilution (0 d); B = 5 d after dilution; C = 10 d after dilution and D = 15 d after dilution.

**Supplementary Figure S2. Number of DNA bands of the soil bacterial community during initial structuring (15 d).** Legends: A = immediately after dilution (0 d); B = 5 d after dilution; C = 10 d after dilution and D = 15 d after dilution. NS = natural soil (control); D1 = dilution 10^-1^; D3 = dilution 10^-3^ and D6 = dilution 10^-6^. Different letters indicate differences between means by the Tukey test (α <0.05).


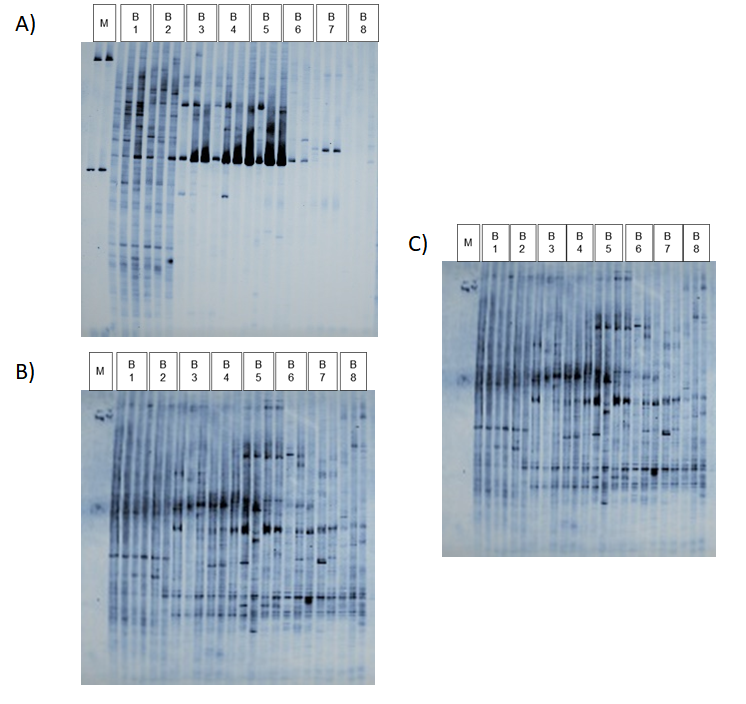


**Supplementary Figure S3.** Bacterial community profiles (bands of 16S RNA gene) obtained by PCR-DGGE. A = 0 d; B = 21 d; and C = 42 d after chlorothalonil and biochar application. M = marker; B1 = natural soil with biochar; B2 = natural soil without biochar; B3 = dilution 10^-1^ with biochar; B4 = dilution 10^-1^ without Biochar; B5 = dilution 10^-3^ with biochar; B6 = dilution 10^-3^ without biochar; B7 = dilution 10^-6^ with biochar; and B8 = dilution 10^-6^ without biochar.

**Supplementary Figure S4. Number of DNA bands of the soil bacterial community after microbial dilution, chlorothalonil and biochar application in the soil (42 d).** Legends: A = 1 d after application; B = 21 d after application and C = 42 d after application. NS = natural soil (control); D1 = dilution 10^-1^; D3 = dilution 10^-3^ and D6 = dilution 10^-6^. Bars followed by different letters indicate a difference between the means by the Tukey test at the significance level of 5.0%.

**Supplementary Table 1. Analysis of similarity (ANOSIM) of the bacterial community profile after microbial dilution, chlorothalonil and biochar application in the soil (42 d). Legends: cluster "C1" = natural soil (with biochar and without biochar); cluster "C2" = dilution 10^-1^ (with biochar and without biochar); cluster "C3" = dilution 10^-3^ (with biochar and without biochar) and cluster "C4" = dilution 10^-6^ (with biochar and without biochar); R _value_ = degree of similarity; Groupings of samples according to degree of similarity: R _values_ > 0.75 indicate difference between samples; 0.50 > R _value_ > 0.75 indicate overlapping separation between samples and R _value_ < 0.50 indicate no significant differences between samples. *Significant (*P _value_ < 0.001*).**

| **Time (d)** | **R *value*** | **C1** | **C2** | **C3** | **C4** |
| --- | --- | --- | --- | --- | --- |
| 1 | **C1** | 0.0000 | 0.8611* | 0.8407* | 0.8741* |
|  | **C2** | - | 0.0000 | 0.3278 | 0.7185* |
|  | **C3** | - | - | 0.0000 | 0.4963* |
|  | **C4** | - | - | - | 0.0000 |
|  | **R *value*** | **C1** | **C2** | **C3** | **C4** |
| 21 | **C1** | 0.0000 | 0.2537 | 0.3056 | 0.4611* |
|  | **C2** | - | 0.0000 | 0.5370* | 0.5870* |
|  | **C3** | - | - | 0.0000 | 0.4463* |
|  | **C4** | - | - | - | 0.0000 |
|  | **R *value*** | **C1** | **C2** | **C3** | **C4** |
| 42 | **C1** | 0.0000 | 0.6296* | 0.8426* | 0.8333* |
|  | **C2** | - | 0.0000 | 0.6167* | 0.5000* |
|  | **C3** | - | - | 0.0000 | 0.6222* |
|  | **C4** | - | - | - | 0.0000 |
